# Inhibitory effects and mechanism of 5-fluorouracil combined with celecoxib on human gastric cancer xenografts in nude mice

**DOI:** 10.3892/etm.2014.2077

**Published:** 2014-11-18

**Authors:** XIAO-QIAN ZHANG, HONG-MEI ZHANG, XIU-E SUN, ZHOU-JIE YUAN, YU-GUANG FENG

**Affiliations:** Department of Gastroenterology, Affiliated Hospital of Weifang Medical University, Weifang, Shandong 261031, P.R. China

**Keywords:** 5-fluorouracil, celecoxib, hypoxia-inducible factor-2α, ATP-binding cassette transporter G2, octamer-binding transcription factor 4, nude mice, gastric cancer

## Abstract

5-Fluorouracil (5-Fu) is one of the most commonly used drugs to treat gastric cancer; however, drug-resistance in cancer cells reduces the efficacy of 5-Fu. Celecoxib may be able to reduce resistance to 5-Fu chemotherapy. The aim of the present study was to investigate the inhibitory effects of a combination of 5-Fu and celecoxib on implanted gastric cancer xenografts in nude mice and to elucidate the underlying mechanism. A tumor-bearing nude mice model was established. The mice were divided into blank control, 5-Fu, celecoxib and combination groups. The weight change and the tumor inhibition rate in each group were calculated. Immunocytochemistry, reverse transcription-polymerase chain reaction and western blotting methods were used to observe hypoxia-inducible factor-2α (HIF-2α), ATP-binding cassette transporter G2 (ABCG2) and octamer-binding transcription factor 4 (Oct-4) expression in the SGC7901 cells. Inhibition of the growth of the implanted gastric cancer was observed in the 5-Fu, celecoxib and combination groups. In the celecoxib and combination treatment groups, the mean tumor mass was significantly less than that in the control group (P<0.05), and the mean tumor mass in the combination treatment group was significantly less than that in the 5-Fu group (P<0.05). The tumor inhibition rates in the 5-Fu, celecoxib and combination groups were 26.36, 59.70 and 88.37%, respectively. The combination group exhibited the highest inhibition rate; the inhibition rates of the combination and celecoxib groups were significantly higher compared with the 5-Fu group (P<0.05). The expression levels of HIF-2, ABCG2 and Oct-4 mRNA and protein were high in the blank control group, and were further increased in the 5-Fu group. However, in the celecoxib and combination groups, the expression levels were lower compared with those in the control group. Significant differences were identified among the 5-Fu, celecoxib and combination groups (P<0.01). Celecoxib has antitumor effects *in vivo*. The mechanism may be associated with the reduced expression of cancer stem cell markers HIF-2α, Oct-4 and ABCG2. 5-Fu and celecoxib have a synergistic antitumor effect. The mechanism associated with the amelioration of resistance to chemotherapy in gastric cancer and the enhancement of the effect of chemotherapy may be via the reduction of the expression of HIF-2α, ABCG2, Oct-4 and other cancer stem cell markers in the tumor tissues.

## Introduction

Chemotherapy is one of the primary treatments for gastric cancer (1). Although many new anticancer drugs and chemotherapies have been introduced, there has been no significant progress in the treatment effect. The main reason is that gastric cancer cells develop multidrug resistance to chemotherapeutic drugs, which significantly limits the application of chemotherapy drugs. 5-Fluorouracil (5-Fu) is an anti-metabolic chemotherapeutic agent. It is the most frequently selected drug in the clinical adjuvant chemotherapy and neoadjuvant chemotherapy of tumors. It inhibits thymidylate synthase and thereby blocks the transformation of deoxyuridylate into deoxythymidylate. It affects DNA synthesis and leads to cell damage and death (2). However, the presence of drug resistance in cancer patients reduces the efficacy of 5-Fu. Celecoxib is a non-steroidal anti-inflammatory drug (NSAID), which is a selective cyclooxygenase-2 (COX-2) inhibitor with anti-inflammatory and analgesic effects (3). According to clinical and experimental studies, celecoxib also has a role in tumor suppression; however, the exact mechanism by which the NSAID acts as a specific antitumor drug is unclear (4–6). Preliminary experiments of the present study indicated that celecoxib can inhibit the proliferation of SGC7901 human gastric cancer cells *in vitro* and may be combined with 5-Fu to reduce the expression of cancer stem cell markers such as hypoxia-inducible factor-2α (HIF-2α), ATP-binding cassette transporter G2 (ABCG2) and octamer-binding transcription factor 4 (Oct-4). On the basis of these previous experiments, the current study used human gastric cancer cells transplanted in nude mice to investigate the inhibitory effects of celecoxib on SGC7901 cell growth *in vivo*. Whether the combination of 5-Fu and celecoxib is able to reduce the expression of stem cell markers HIF-2α, ABCG2 and Oct-4 in human gastric carcinoma tumors transplanted into nude mice and improve the resistance to 5-Fu chemotherapy was also examined.

## Materials and methods

### Materials

The human gastric cancer cell line SGC7901 was given as a gift from Professor Yuguang Feng of the Affiliated Hospital of Weifang Medical College (Weifang, China). 5-Fu was acquired from Jiangsu Zhenguo Pharmaceutical Co., Ltd. (Nantong, China). Celecoxib was purchased from Pfizer (New York, NY, USA). Rabbit anti-HIF-2α, Oct-4 and ABCG2 polyclonal antibodies were from Abcam (HIF-2α, ab73895; Oct-4, ab18976; ABCG2, ab186770; Cambridge, MA, USA). Immunohistochemistry kits (SP-9000) were purchased from Zhongshan Golden Bridge Biotechnology Co., Ltd. (Beijing, China). TRIzol reagent was from Invitrogen Life Technologies, and the reverse transcription (RT) and polymerase chain reaction (PCR) kits were purchased from Fermentas (Thermo Fisher Scientific). The protein extraction kit was from Biyuntian Co. (Jiangsu, China). The western blot enhanced chemiluminescence kit was from Thermo Fisher Scientific. The 28 male nude mice (BALB/c nu/nu; age, 5–6 weeks) were purchased from Beijing Weitong Lihua Laboratory Animal Technology Co., Ltd. (Beijing, China). The mice weighed 18–22 g and were raised in a specific pathogen-free environment.

### Establishment of a tumor-bearing nude mice model

A total of 28 male mice (BALB/c nu/nu) aged 5–6 weeks and weighing 18–22 g were used in the experiment. SGC7901 human gastric cancer cells in the logarithmic growth phase were used to create a cell suspension with a concentration of 1×10^7^/ml. Under sterile conditions, 0.2 ml cell suspension was inoculated subcutaneously into the nude mice, which were continuously fed for two weeks to establish the nude mouse model. The inoculated mice were randomly divided into four groups, with seven mice in each group; the body weight difference between groups was not significant. In the blank control group, an intraperitoneal injection of saline (10 ml/kg) was performed every other day. In experimental group 1 (the 5-Fu group), an intraperitoneal injection of 5-Fu (60 mg/kg) was administered every other day. In experimental group 2 (the celecoxib group), an intraperitoneal injection of celecoxib (30 mg/kg) was given every other day. In experimental group 3 (the combination group), celecoxib (30 mg/kg) and 5-Fu (60 mg/kg) were administered by injection every other day. All treatments were continued for 2 weeks. The diet, activity, urine and tumor growth of the nude mice were observed every day. At the end of the experiment, the mice were weighed and the tumor size was measured. Mice were sacrificed by cervical dislocation, the tumor was stripped with scissors and the tumor weight was documented. The procedures carried out in the present study were approved by the Medical Ethics Committee of the Affiliated Hospital of Weifang Medical University (Weifang, China).

### Body weight change and calculation of tumor inhibition rate

On the day after the final administration, the animals were sacrificed by cervical dislocation, the tumor tissue was excised and the tumor mass was weighed using electronic scales. The inhibition rate in each group was calculated according to the following formula: Inhibition rate (%) = (average tumor weight in the control group - average tumor weight in experimental group)/average tumor weight in control group × 100. The short and long diameters of the tumor were measured and the tumor volume was calculated using the following formula: Tumor volume (V) = (L × S^2^)/2, where L is the long diameter and S is the short diameter of the tumor.

### Expression of HIF-2α, ABCG2 and Oct-4 mRNA and protein

#### Immunohistochemistry

Mice xenograft specimens from each group were cut (5 μm) and fixed with 10% formalin. The sections were paraffin-embedded, stained with hematoxylin and eosin and observed under a light microscope. The immunohistochemical staining of tumor tissue from each group was conducted using an SP-9000 kit according to the instructions of the manufacturer. HIF-2α, ABCG2 and Oct-4 antibody staining was carried out (dilution 1:50) following which the tissues were placed in a 4°C refrigerator overnight. 3,3′-Diaminobenzidine color rendering, dehydration, transparency were performed and the tissues were mounted with neutral gum. Phosphate-buffered saline replaced the primary antibody to act as the negative control. Staining for HIF-2α, Oct-4 and ABCG2 was considered to be positive when brown particles were observed within the cytoplasm.

#### RT-PCR

The TRIzol method was used for extraction of total RNA from the tumor tissue. The RNA was dissolved in 30 μl diethylpyrocarbonate-treated water. First-strand cDNA was reversely synthesized, using an RT reaction system (20 μl) as follows: 9 μl deionized water with no RNA enzyme, 2 μl template RNA, 181 μl Oligo (dT), 4 μl 5× reaction buffer, 1 μl RNase inhibitor (20 U/μl), 2 μl dNTP mix (10 mmol/l) and 1 μl M-MuLV RT. The reaction conditions were as follows: 70°C for 5 min and then placed on ice; 37°C for 5 min; 37°C for 60 min; 70°C for 10 min and then placed on ice for subsequent testing or preservation at −150°C.

The primers used were HIF-2α, forward: 5′-CTTGGA GGGTTTCATTGCTGTGGT-3′ and reverse: 5′-GTGAAG TCAAAGATGCTGTGTCCT-3′, with a product length of 123 bp; ABCG2, forward: 5′-CCCTTATGATGG TGGCTTATTC-3′ and reverse: 5′-GTGAGATTGACC AACAGA CCAT-3′, with a product length of 132 bp; Oct-4, forward: 5′-CCCGAAAGAGAAAGCGAACC-3′ and reverse: 5′-CAGAACCACACTCGGACCAC-3′, with a product length of 151 bp; and GAPDH, forward: 5′-GCACCACCA ACTGCTTAGCAC-3′ and reverse: 5′-GCAGCGCCA GTAGAGGCAGG-3′, with a product length of 143 bp. In the 50-μl PCR reaction system, 1 μl template cDNA, 1 μl each of upstream and downstream primers, 1 μl Taq DNA polymerase, 5 μl dNTPs (2 mmol/l), 2 μl MgCl_2_ (25 mmol/l), 5 μl 10× PCR buffer and 34 μl ddH_2_O were maintained at 94°C for 5 min; 94°C for 30 sec; 50°C for 30 sec and 72°C for 60 sec for 40 cycles. Extension was carried out at 72°C for 10 min and 4°C for +∞. Then, 1.5% agarose gel electrophoresis was used for identification of the product. A digital gel imaging system was used to capture images and the optical density of the amplification products was analyzed. Through analysis of the HIF-2α, ABCG2 and Oct-4 mRNA and GAPDH optical density values, the expression levels of HIF-2α, ABCG2 and Oct-4 mRNA were evaluated.

#### Western blotting

The total protein of the tumor tissue was extracted. The bicinchoninic acid assay method was used to determine the protein concentration. SDS-PAGE gel electrophoresis was then conducted. The proteins were placed on a cellulose membrane and sealed with 5% skimmed milk powder at 37°C for 2 h. HIF-2α, ABCG2 and Oct-4 and β-actin antibodies were added (dilution, 1:1,000), and the membrane was incubated overnight at 4°C. After washing the membrane with Tris-buffered saline and Tween 20, the secondary antibody horseradish peroxidase-labeled anti-IgG (Invitrogen Life Technologies) was added and the membrane was incubated for a further 2 h at 37°C. After washing the membrane, the electroluminescence (ECL) reagent was added. X-ray film exposure, developing and fixing were performed. LabWorks analysis software, version 4.5 (Ultra-Violet Products, Inc., Upland, CA, USA) was used to measure the absorbance value of the western blotted strip; the ratio of the absorbance value of the protein of interest to that of β-actin was considered to indicate the relative content of HIF-2, ABCG2 and Oct-4.

### Statistical analysis

Data were analyzed using the SPSS statistical package, version 17.0 (SPSS, Inc., Chicago, IL, USA). Quantitative data are expressed as the mean ± standard deviation. Quantitative data were compared between groups using a Student’s t-test and analysis of variance (ANOVA). P<0.05 was considered to indicate a statistically significant difference.

## Results

### Weight change and tumor inhibition rate

**Figure 1:**
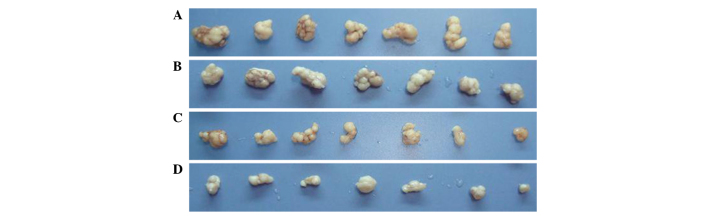
Tumor volume changes in xenografted nude mice following treatment. (A) control, (B) 5-fluorouracil, (C) celecoxib and (D) combination groups..

The formation of nodules was observed in the 28 nude mice 14 days after gastric cancer cell inoculation; all grew into a tumor. The average volumes of the tumor mass in the four groups were not significantly different prior to the administration of the various treatments (P>0.05). However, 15 days after the treatment was initiated (1 day after the final day of treatment), the average volume of the tumor mass in the 5-Fu group was less than that in the control group, but the difference was not significant (P>0.05). The average volumes of the tumor mass in the celecoxib group and the combined group were significantly lower than that in the control group (P<0.05), and the average volume of the tumor mass in the combined treatment group was significantly less than the volume in the 5-Fu group (P<005). Similar results were obtained for the tumor weight. The mean tumor weight in the 5-Fu group was not statistically significant different from that in the control group (P>0.05). The mean tumor weights in the celecoxib and combined treatment groups were significantly lower than that in the control group (P<0.05), and the mean tumor weight in the combined treatment group was significantly less than the mean weight in the 5-Fu group (P<0.05). The tumor inhibition rates in the 5-Fu, celecoxib and combination groups were 26.36, 59.70 and 88.37%, respectively. A statistically significant difference was identified between the combination group and the 5-Fu and celecoxib groups (Table I and Fig. 1).

### HIF-2α, ABCG2 and Oct-4 protein expression in tumor tissue evaluated by immunohistochemical staining

The positive expression of HIF-2α, ABCG2 and Oct-4 proteins was identified as brown granular staining. The proteins were mainly dispersed throughout the cytoplasm and along the nuclear membrane border in a linearly distributed manner, and a strong positive reaction was observed in the nucleus.

**Figure 2:**
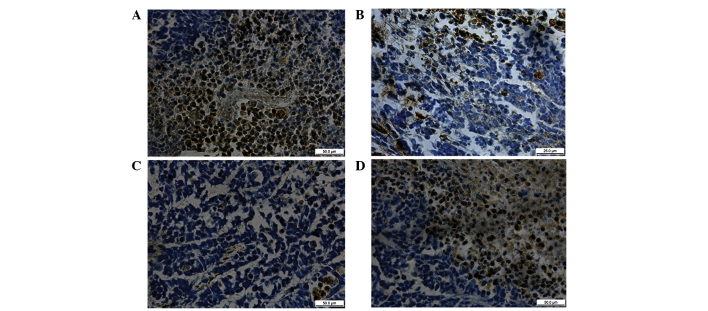
Expression of HIF-2α protein in xenograft tissue detected by immunohistochemistry (magnification, ×400). (A) 5-Fu group, (B) celecoxib group, (C) combination group and (D) control group. HIF-, hypoxia-inducible factor-2α; 5-Fu, 5-fluorouracil.

**Figure 3:**
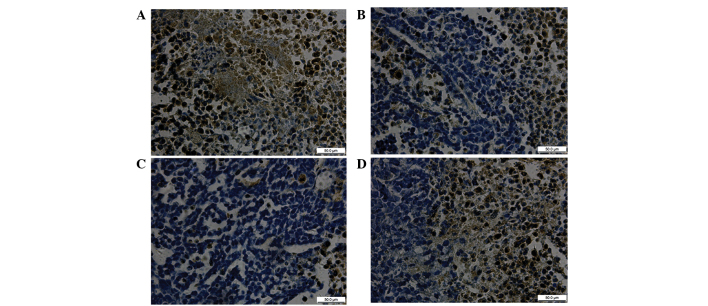
Expression of ABCG2 protein in xenograft tissue detected by immunohistochemistry (magnification, ×400). (A) 5-Fu group, (B) celecoxib group, (C) combination group and (D) control group). 5-Fu, 5-fluorouracil.

**Figure 4:**
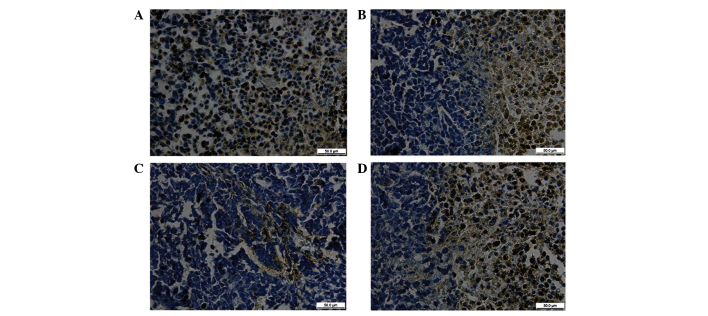
Expression of Oct-4 protein in xenograft tissue detected by immunohistochemistry (magnification, ×400). (A) 5-Fu group, (B) celecoxib group, (C) combination group and (D) control group. Oct-4, octamer-binding transcription factor 4; 5-Fu, 5-fluorouracil.

For the four groups of nude mice after 14 days, the expression levels of HIF, ABCG2 and Oct-4 proteins were highest in the tumor tissue of the 5-Fu group, followed by the blank control group, and the expression levels of the three proteins in the combined and celecoxib groups were significantly reduced (Figs. 2–4).

### HIF-2α, ABCG2 and Oct-4 mRNA expression in tumor tissue evaluated by RT-PCR

**Figure 5:**
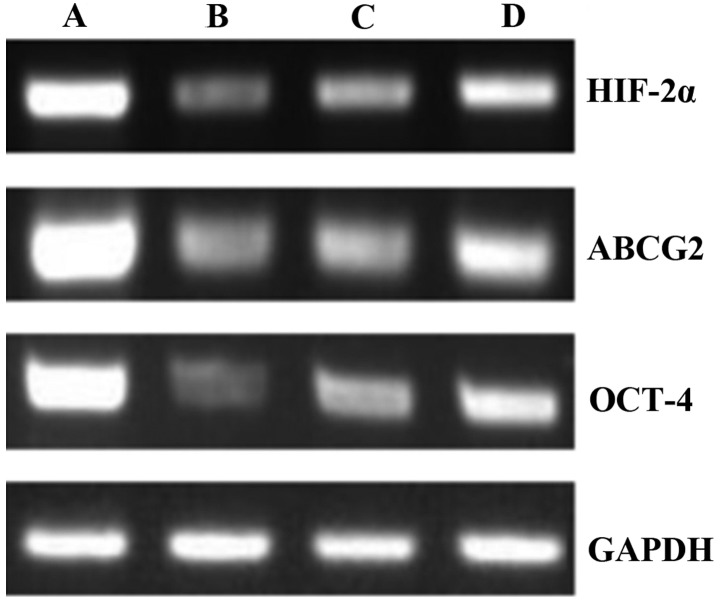
HIF-2α, ABCG2 and Oct-4 mRNA expression in tumor tissues of each group detected by reverse transcription polymerase chain reaction. Lane A, control group; lane B, 5-Fu group; lane C, celecoxib group; lane D, combination group. HIF-2α, hypoxia-inducible factor-2α; Oct-4, octamer-binding transcription factor 4; 5-Fu, 5-fluorouracil.

**Figure 6:**
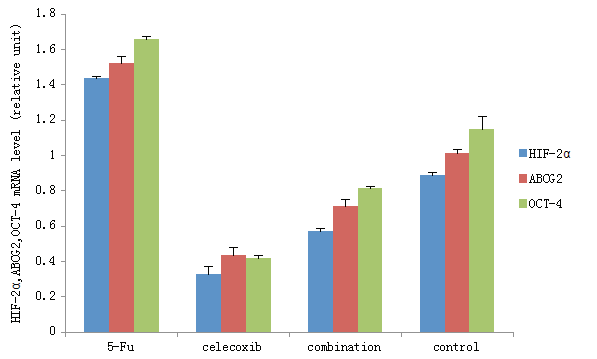
HIF-2α, ABCG2 and Oct-4 mRNA expression levels in tumor tissues of each group detected by reverse transcription polymerase chain reaction. HIF-2α, hypoxia-inducible factor-2α; Oct-4, octamer-binding transcription factor 4. Pairwise comparison revealed significant differences between the expression levels of HIF-2α, ABCG2 and Oct-4 in the different groups (P<0.01).

The RT-PCR technique was used in the four groups of nude mice to detect the mRNA expression of HIF-2α, ABCG2 and Oct-4 in the tumor tissue after 14 days of treatment. In the control group, which received saline every other day, HIF-2α, ABCG2 and Oct-4 mRNA expression was observed at high levels. In the 5-Fu group, the levels of HIF-2α, ABCG2 and Oct-4 mRNA expression were increased by the injection of 5-Fu every other day. In the celecoxib and combined treatment groups, the HIF-2α, ABCG2 and Oct-4 mRNA expression levels were lower than those in the 5-Fu group. The celecoxib and combination treatment groups showed a significant difference from the control group when a pairwise comparison was performed (P<0.01; Figs. 5 and 6).

### HIF-2α, ABCG2 and Oct-4 protein expression in tumor tissue evaluated by western blotting

**Figure 7:**
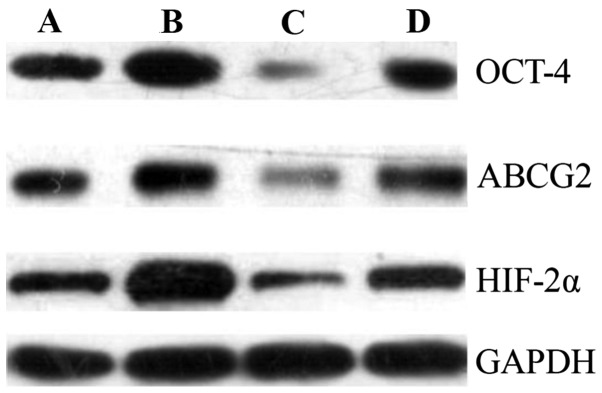
HIF-2α, ABCG2 and Oct-4 protein expression in tumors of each group detected by western blot analysis. Lane A, control group; lane B, 5-Fu group; lane C, celecoxib group; and lane D, combination group. HIF-2α, hypoxia-inducible factor-2α; Oct-4, octamer-binding transcription factor 4.

**Figure 8:**
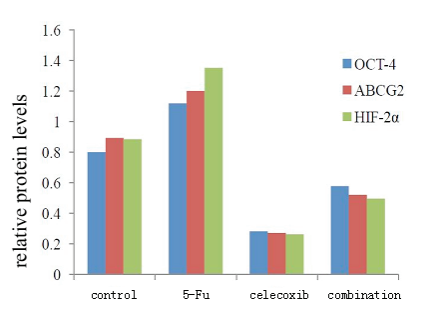
Expression of HIF-2α, ABCG2 and Oct-4 proteins in tumors of each group as determined by western blot analysis. A, control group; B, 5-Fu group; C, celecoxib group; and D, combination group. HIF-2α, hypoxia-inducible factor-2α; Oct-4, octamer-binding transcription factor 4. Pairwise comparison of each group was performed; the differences in the expression levels of HIF-2α, ABCG2 and Oct-4 were statistically significant between all groups (P<0.01).

The western blotting technique was used to detect the protein expression of HIF-2α, ABCG2 and Oct-4 in the tumor tissue after 14 days of treatment. The results showed that the HIF-2, ABCG2 and Oct-4 protein expression levels in the tumor tissue were high in the control group, and were further increased in the 5-Fu group. The HIF-2α, ABCG2 and Oct-4 protein expression in the tumor tissue was significantly decreased in the celecoxib and combination treatment groups compared with the control and 5-Fu groups. For all four groups, a pairwise comparison was performed (Figs. 7 and 8). The protein expression levels of HIF-2α, ABCG2 and OCT-4 within each group were progressively lower from the 5-Fu group (highest level), to the control group, and to the celecoxib and combination groups (both the lowest levels; all P<0.05).

## Discussion

Gastric cancer is an disease that is seriously harmful to human health, as it has a high morbidity and mortality. Surgery remains the only mean possible to cure gastric cancer, but in approximately two-thirds of cases, the patient’s condition has reached advanced gastric cancer at the time of diagnosis (7,8). It has a high rate of recurrence and metastasis. Chemotherapy is a primary treatment means for gastric cancer (9). Although new anticancer drugs and chemotherapies have been introduced, there has been no significant progress in the effectiveness of treatment. This is primarily due to gastric cancer cells developing multidrug resistance to chemotherapeutic drugs, which limits the application of chemotherapy drugs. 5-Fu is one of the most frequently selected drugs in clinical adjuvant chemotherapy and neoadjuvant chemotherapy. It acts as an inhibitor of thymidylate synthase, and blocks the transformation of deoxyuridylate into deoxythymidylate, affects DNA synthesis and leads to cell damage and death (10). Apoptosis is one of the anti-tumor mechanisms of 5-Fu (11). In addition, it is a cell cycle-specific drug; it can inhibit each stage of the cell cycle, but has its optimum effects on cells in the S phase (12). However, resistance in cancer patients reduces the practical effect of 5-Fu. The present study revealed that the tumor inhibition rate of 5-Fu was only 26.36% in gastric xenografts in nude mice. When compared with the saline-treated control group, the difference in tumor weight was not statistically significant. The identification of novel methods to attenuate tumor resistance to chemotherapy and to enhance the effect of chemotherapy is necessary.

Previous studies have suggested that a very small amount of tumor tissue has unlimited self-renewal capacity and multi-cell proliferative potential, due to the presence of cancer stem cells. These are considered to be the root cause of metastasis, recurrence, drug resistance and chemotherapy resistance (13,14). However, at present, since many cancer stem cell markers have not yet been determined, it is not possible to directly isolate and identify the stem cells. HIFs are closely associated with the malignant phenotype of tumor angiogenesis, invasion, metastasis and chemotherapy resistance (15). For cancer stem cells, the association with HIF-2α is closer than that with HIF-1α. HIF-2α can regulate a variety of stem cell-related pathways to maintain the stem cell phenotype and allow the transformation of non-stem cells into a stem cell phenotype. ABCG2 is a member of the ABC transporter super family, which can cause the efflux of a variety of chemotherapy drugs. Its high level of expression has been found to be a significant cause of multidrug resistance (16). This previous study identified that a plurality of stem cells highly expressed ABCG2. ABCG2 is a direct target gene of HIF-2α. The high expression of components of the HIF-2α-ABCG2 pathway leads to MDR in cancer stem cells (17). Oct-4 is a member of the POU family of transcription factors and totipotent or pluripotent stem cell markers. Previous studies have suggested that Oct-4 may be closely associated with tumor stem cells (18,19). Covello *et al* (20) reported that Oct-4 is a direct target gene of HIF-2α. Hypoxia can activate the HIF-2α-Oct-4 pathway to maintain the tumor stem cell phenotype. Dallas *et al* (21) demonstrated that compared with their parental cells, 5-Fu-resistant colon cancer cells (HT29/5Fu-R) highly expressed the stem cell phenotype (CD133^+^/CD44^+^), indicating that a 5-Fu insensitive subpopulation of cancer stem cells is the source of resistance to chemotherapy. The preliminary results of the present study demonstrated that under hypoxic conditions, when 5-Fu was used to treat human gastric cancer cell lines, the expression of cancer stem cell markers HIF-2α and ABCG2 increased (22). In the present study, following the intraperitoneal injection of 5-Fu into gastric cancer xenografts in nude mice, the HIF-2α, ABCG2 and Oct-4 mRNA and protein levels increased, indicating that the gastric cancer cells were exhibiting chemotherapy resistance to 5-Fu. This chemoresistance may be associated with the high expression of cancer stem cell markers HIF-2α, ABCG2 and Oct-4 and tumor stem cell promotion.

Celecoxib is a selective COX-2 inhibitor that has anti-inflammatory and analgesic effects. Clinically, it is used for the treatment of acute and chronic osteoarthritis and rheumatoid arthritis. Compared with conventional NSAIDs, it has significantly reduced gastrointestinal side-effects. Clinical and experimental studies have shown that celecoxib also plays a role in tumor suppression. Steinbach *et al* (23) performed a double-blind, placebo-controlled clinical trial, which showed that celecoxib inhibited the formation of familial adenomatous polyposis. Animal experiments have shown that celecoxib has preventive and suppressive effects on gastric cancer (24,25). In the present study, the tumor inhibition rates in the 5-Fu, celecoxib and combination groups were 26.36, 59.70 and 88.37%, respectively. The inhibition rate in the combination group was statistically significantly different from those in the 5-Fu and celecoxib groups. This indicates that celecoxib is able to inhibit tumor growth *in vivo,* specifically, in gastric cancer transplants in nude mice. The inhibition rate in the combination treatment group was significantly enhanced compared with that in the 5-Fu group. Celecoxib and 5-Fu exhibited a synergistic effect in the treatment of gastric cancer. However, the specific antitumor mechanism of NSAIDs remains unclear. Previous cell and animal experiments have shown that NSAIDs inhibit COX-2 activity to reduce the synthesis of prostaglandin E2, thereby inducing tumor cell apoptosis (26). However, Ding *et al* found in a premalignant and malignant oral mucosal cell culture model, that the potential anticancer and apoptosis-inducing effects of celecoxib occurred via a mechanism that was independent of COX pathways (27). Numerous other studies have indicated that the NSAID celecoxib can promote the apoptosis of tumor cells and achieve an antitumor effect via non-COX-2 dependent pathways. The mechanism by which COX-2 inhibitors promote tumor cell apoptosis has been indicated to be achieved via regulation of the mRNA and protein expression of p21, Fas, Akt, GSK3β, FKHR, caspase-9, bcl-2/bax, p53 and survivin genes (28–33). The present study found that the NSAID celecoxib reduces HIF-2α, Oct-4 and ABCG2 mRNA and protein expression in gastric cancer tissues implanted in nude mice. This result indicates that in addition to acting via an apoptosis-promoting pathway, celecoxib may achieve antitumor effects by reducing the expression of the cancer stem cell markers HIF-2α, Oct-4 and ABCG2.

In conclusion, HIF-2α, ABCG2 and Oct-4 mRNA and protein expression levels were significantly increased in the tumor tissues of the 5-Fu group; this may be a due to the tumor cells having resistance to 5-Fu. However, when 5-Fu and celecoxib were used together, compared with 5-Fu used alone, the HIF-2α, ABCG2 and Oct-4 mRNA and protein expression levels were significantly lower, and the difference was statistically significant. This showed that the combined use of 5-Fu and celecoxib is able to attenuate the resistance to chemotherapy in gastric cancer and enhance the effect of chemotherapy by reducing the expression of HIF-2α, ABCG2 and Oct-4 and cancer stem cells in tumor tissue xenografts in nude mice.

## Figures and Tables

**Table I:** Volume and body weight changes in each group of xenografted nude mice following treatment.

Group	Tumor volume (cm^3^)	Tumor weight (g)	Inhibition rate (%)
Control	0.691±0.197	1.287±0.274	-
5-Fu	0.586±0.135	0.949±0.185	26.36
Celecoxib	0.255±0.035^a^,^b^	0.523±0.146^a^,^b^	59.70^b^
Combination	0.101±0.031^a^,^b^	0.153±0.023^a^,^b^	88.37^b^

Values presented are the mean ± standard deviation.

a P<0.05 vs. the control group;

b P<0.05 vs. the 5-Fu group.

5-Fu, 5-fluorouracil.
